# Risk factors for therapy failure after incision and drainage alone for perianal abscesses in children

**DOI:** 10.3389/fped.2024.1342892

**Published:** 2024-02-08

**Authors:** CaiLin Ding, YaJun Chen, JiaYu Yan, Kai Wang, Sarah Siyin Tan

**Affiliations:** Department of General Surgery, Beijing Children’s Hospital, National Center for Children’s Health, Capital Medical University, Beijing, China

**Keywords:** perianal abscesses, recurrence, fistula-in-ano, risk factors, prognosis

## Abstract

**Background:**

It is well known that recurrent perianal abscesses (PAs) and fistula-in-ano (FIA) are the main causes of therapy failure following incision and drainage (I&D) for PAs. But few studies have focused on the risk factors for therapy failure after I&D for PAs in children. In this study, we retrospectively examine the risk factors for therapy failure after I&D for PAs in children in a pediatric tertiary care institution.

**Methods:**

A retrospective review of all outpatient children with PA treated by I&D at Beijing Children's Hospital between January 2021 and December 2022 was performed. A follow-up was conducted in October 2023. Patients with other predisposing factors for perianal infection, such as inflammatory bowel disease, hematologic tumor, and anorectal surgery, were excluded from this study. Logistic regression yielding odds ratios (ORs) was used to assess the significance of variables for therapy failure.

**Results:**

Of 160 children initially identified, follow-up was available for 146, with a total of 172 treatments. A total of 91% of children were male. The median (interquartile range) age at I&D was 2 (1, 15) months. The median follow-up duration was 20 (14, 25) months. Therapy failure occurred in 25 (15%) treatments performed for the prevention of recurrence of PA and in 35 (20%) treatments for the prevention of development of FIA. In the univariate analysis, a history of PA (*P* = 0.001), history of I&D (*P* = 0.014), and multilocal occurrence (*P* = 0.003) were associated with therapy failure. A sitz bath after I&D (*P* = 0.016) and regular cleaning of the wound after I&D (*P* = 0.024) were associated with therapy success. In the multivariate analysis, a history of PA (*P* = 0.015, OR = 3.374) and multilocal occurrence (*P* = 0.012, OR = 4.649) were independently associated with therapy failure. Regular cleaning of the wound (*P* = 0.017, OR = 0.341) and sitz bath (*P* = 0.001, OR = 0.128) after I&D were independently associated with therapy success.

**Conclusions:**

A history of PA and multilocal occurrence were predictor factors for therapy failure before I&D. Regular cleaning of the wound and sitz bath after I&D were protective factors for therapy success. Therefore, regular cleaning of the wound and sitz bath after I&D should be emphasized in all children with PAs, especially in those with a history of PA and multilocal occurrence.

## 1 Introduction

A perianal abscess (PA) is a common anorectal problem affecting both children and adults, which is defined as the accumulation of pus in the perianal region (1–5). The rate of incidence of PA is estimated to be between 0.5% and 4.3% for all infants (4, 6). Most of these abscesses are diagnosed before the age of 2 years and mainly occur in otherwise healthy children, especially in robust breastfed infants, with a male predominance in 90%–100% of cases (4, 6–15). Different from adults, PA is rarely accompanied by disseminated systemic infection or sepsis (e.g., fever, general discomfort) in children (3, 7, 13, 15–17). The optimal management of PA in children remains controversial and differs from therapy recommendations in practice guidelines for adults (3, 4, 16, 18–20). Numerous methods are available for the treatment of PA in children that range from conservative treatment to surgery, but the choice of the treatment method mostly depends on the personal experiences and judgments of doctors, as well as the predilection of parents, rather than established guidelines (1–4, 6, 10, 19, 21–25).

Incision and drainage (I&D) is the most common and parent-tolerated surgical treatment for PA in children (1, 2, 6, 7, 10, 11, 23, 26–32). Some of the advantages of I&D are its simplicity, effectiveness, rapid symptom relief, cost-efficiency, obviating the need for hospitalization, and its execution in an outpatient clinic (3, 4, 11, 29). Another benefit of I&D is the avoidance of general anesthesia, a method which is a concern for many parents (3, 4, 7, 10, 11, 28, 32). However, it is reported that 13%–85% of pediatric patients develop recurrent PA and/or a fistula-in-ano (FIA) that requires further treatment after I&D (4, 6, 9, 10, 14, 22, 27, 29, 31, 33).

Therefore, before attempting I&D, it would be clinically useful to identify factors predisposing patients to therapy failure. To the best of our knowledge, no research has investigated the risk factors for therapy failure after I&D for PAs in children. Thus, in this study, we aim to fill this research gap.

## 2 Patients and methods

The study was approved by the Ethics Committee of Beijing Children's Hospital, Capital Medical University [study ID: (2023)-E-161-R].

### 2.1 Patients

The patients who met the inclusion criteria for the period between January 2021 and December 2022 at Beijing Children's Hospital were included in this study. Data for this retrospective study were gathered by reviewing the medical records in the data bank of outpatients. The inclusion criteria were (1) outpatient diagnosis of PA and (2) patients who underwent I&D for PAs at our center. Patients who presented with a combination of other underlying predisposing conditions, such as inflammatory bowel disease, immune deficiency, malignant tumors, FIA, and anorectal malformation, had a history of trauma or perianal foreign body before PA, or had a history of anorectal surgery, such as Hirschsprung's disease, were excluded. Demographic information, clinical and laboratory data, and pre-and postoperative medical treatment details were gathered from the patients.

### 2.2 Methods

#### 2.2.1 Treatment strategy

I&D for PA was performed in the outpatient setting under local anesthesia. A local anesthetic ointment containing lidocaine and prilocaine was applied for 1 h before I&D. A small incision was made through the highest site of the abscess with a scalpel, ensuring complete drainage of pus and necrotic tissue. The pockets within the PA cavity were delicately broken via exploration. After pus and necrotic tissue were completely removed, a Vaseline gauze was stuffed into the PA. Finally, a sterilized dressing was used to cover the wound. No bacterial culture was carried out, and no antibiotics were routinely used after I&D.

After I&D, a standard postoperative treatment protocol was recommended for the parents of children to follow, including keeping the perianal area clean and dry, making dressing changes, asking their children to take sitz baths or use a wet compress, and applying ointment. Regular cleaning of the wound was also strongly recommended, either by the outpatient clinic or by using a cotton swab at home after I&D every day or every 3 days until the wound healed. If patients chose to come to the outpatient clinic, the dressing changes included evaluating the wound and then cleaning and sterilizing it to achieve full drainage. The use of silver ion dressings stuffed into the PA, or alternatively, keeping the wound open and exposed, depended on the nature of the wound. If a patient had loose stools or watery diarrhea, the milk formula and medication were changed to maintain formed stools, such as montmorillonite powder and probiotics. For prolonged diarrhea, lactose intolerance or some kind of allergy was considered. If premature healing of the skin over the PA caused recurrence, the PA was incised again and managed in the same way as stated previously. All surgeries were performed by surgeons from the same surgical team.

#### 2.2.2 Follow-up

The primary endpoint of this study was recurrent PA or formation of FIA. The secondary endpoint was unscheduled repeat outpatient visits or other complaints. Therapy failure was defined as recurrent PA or formation of FIA. Recurrent PA was defined as a PA that developed at the same location after wound healing of I&D. FIA was defined as the presence of a hole with or without pus drainage at the site of the anus, persisting for more than 3 weeks after I&D (34, 35). A history of PA was defined as patients who were required to undergo I&D for PAs at our center because of a recurrence of PA that had been successfully treated with conservative treatment at the same location. Regular cleaning of the wound was defined as cleaning of the wound at the outpatient clinic or using a cotton swab at home after I&D every day or every 3 days for 1 week. Prognostic information after the performance of I&D was collected via telephone interviews and supplemented with outpatient records. A survey of risk factors was performed by using online structured questionnaires and supplemented by telephone interviews, Factors such as abscess recurrence, fistula formation, and subsequent surgery for recurrence were analyzed.

#### 2.2.3 Statistical analyses

The original data were organized using Microsoft Excel, and data analysis was performed using SPSS software (version 24.0). A Kolmogorov–Smirnov test was used for studying the pattern of distribution of the quantitative variables. When the quantitative variables were normally distributed, they were expressed as mean ± standard deviation; if otherwise, they were expressed as median and interquartile range (Q1, Q3). The qualitative variables were expressed as counts and percentages. An independent-sample *t*-test was used to make comparisons between groups for normally distributed data. A Mann–Whitney *U* test was used for comparing non-normally distributed data. Qualitative data were represented by count (%), and the chi-square test was used for making a comparison between groups. Logistic regression yielding odds ratios (ORs) was used to assess the significance of the variables for therapy failure. Significant differences were defined as a *P*-value < 0.05.

## 3 Results

**Figure 1:**
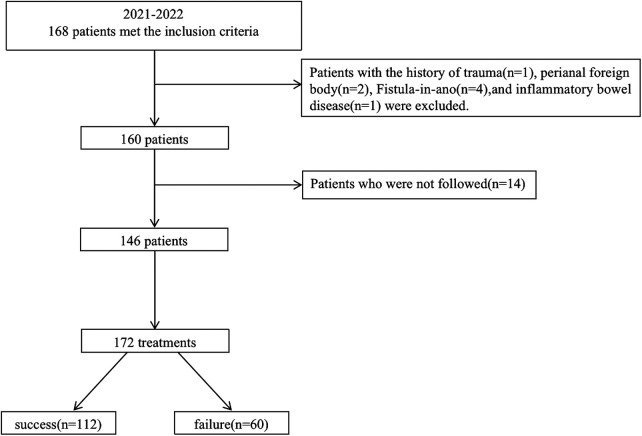
A flowchart of patient inclusion.

A total of 160 children were treated using I&D in the outpatient setting at Beijing Children's Hospital from January 2021 to December 2022. Treatment follow-up was not available for 14 patients (9%), 2 had incorrect contact information, and the parents of 12 children did not give their consent for follow-up. The research flow chart is shown in Figure 1. Fourteen patients underwent a second I&D and 6 patients underwent a third I&D. A total of 172 treatments in 146 patients were considered eligible. The median age at I&D was 2 (1, 15) months. There were 20 patients who had the results of blood routine before I&D, in which 14 (70%) patients had an elevated C-reactive protein and/or white blood count. There were 18 patients who had the results of B-mode ultrasonography before I&D. The median follow-up duration was 20 (14, 25) months. Therapy failure occurred in 25 (15%) treatments performed for the prevention of recurrence of PA and 35 (20%) treatments for the prevention of the development of FIA. There were 16 unscheduled repeat outpatient visits because of a premature healing of the skin over the PA. There were no other complaints. The details are given in Table 1.

**Table 1:** Patient and disease characteristics of 172 I&D procedures.

Items	*n* (%)	Q1, Q3
Cases	146	
No. of I&D		
1	126 (86)	
2	14 (10)	
3	6 (4)	
Total no. of I&D	172	
Male	157 (91)	
Female	15 (9)	
Age at ID	2	1, 15
<3	89 (52)	
3–6 months	15 (8)	
6–12 months	22 (13)	
1–2 years old	12 (7)	
>2 years old	34 (20)	
Perianal abscesses characteristics		
No. of lesions		
1	148 (86)	
2	21 (12)	
3	3 (2)	
Localization at the lithotomy position		
Lateral	120 (70)	
Ventral	25 (14)	
Dorsal	27 (16)	
Length of follow-up (month)	20	14, 25
Success	112 (65)	
Failure	60 (35)	

A comparison of clinical data between the success and the failure group showed no significant difference in the locations of PA, age at I&D, follow-up duration and percentage of migration background, presence of fever, diarrhea, conservative treatment before I&D, and antibiotics in the perioperative period (*P* > 0.05). However, in the failure group, there was a higher male to female ratio, higher percentage of multilocal occurrence, and higher history of PA and history of I&D than in the success group (*P* < 0.05). The percentage of regular cleaning of the wound and sitz bath after I&D was higher in the success group (*P* < 0.05). The details are given in Table 2.

**Table 2:** Patient and disease characteristics related to success and failure therapy.

Items [*n* (%)]	Failure (*n* = 60)	Success (*n* = 112)	*χ* ^2^	*P*
Sex			8.803	0.003
Male	60 (100%)	97 (87)		
Female	0 (0)	15 (13)		
Age^a^	2 (1, 11)	2 (1, 15)	*N* ^b^	0.944
>2 years old			0.377	0.539
Yes	10 (17)	23 (21)		
No	50 (83)	89 (79)		
>1 year old			1.723	0.189
Yes	14 (23)	40 (36)		
No	46 (77)	82 (67)		
Follow-up duration (months)^a^	20 (14, 26)	20 (14, 25)	*N* ^b^	0.761
Migration background			2.062	0.151
Yes	15 (25)	40 (36)		
No	45 (75)	7 (64)		
History of PA			23.783	0.001
Yes	24 (40)	10 (9)		
No	36 (60)	102 (91)		
History of I&D			7.409	0.006
Yes	8 (13)	3 (3)		
No	52 (87)	109 (97)		
Fever			0.427	0.514
Yes	6 (10)	8 (7)		
No	54 (90)	104 (93)		
Diarrhea			3.205	0.073
Yes	47 (78)	73 (65)		
No	13 (22)	39 (35)		
Lateral distribution of a PA at the lithotomy position				
Yes	45 (75)	75 (67)	1.196	0.274
No	15 (25)	37 (33)		
Localization of a PA at the lithotomy position			2.281	0.320
Lateral	45 (75)	75 (67)		
Ventral	9 (15)	16 (14)		
Dorsal	6 (10)	21 (19)		
Multilocal			9.365	0.002
Yes	15 (25)	9 (8)		
No	45 (75)	103 (92)		
Antibiotics in the perioperative period, *n*			1.32	0.251
Yes	9 (15)	25 (22)		
No	51 (85)	87 (78)		
Conservative treatment before I&D, *n*			0.144	0.704
Yes	21 (35)	36 (32)		
No	39 (65)	76 (68)		
Regular cleaning of the wound after I&D			4.198	0.040
Yes	11 (22)	37 (33)		
No	49 (78)	75 (67)		
Sitz bath after I&D			7.261	0.007
Yes	8 (13)	34 (30)		
No	52 (87)	78 (70)		

^a^ Median (Q1, Q3).

^b^ Mann–Whitney *U* test.

Univariate and multivariate analyses for risk factors for therapy failure are provided in Table 3. In the univariate analysis, a history of PA (*P* = 0.001), history of I&D (*P* = 0.014), multilocal occurrence (*P* = 0.003), sitz bath after I&D (*P* = 0.016), and regular cleaning of the wound after I&D (*P* = 0.024) were associated with therapy failure. In the multivariate analysis, a history of PA (*P* = 0.015, OR = 3.374) and multilocal occurrence (*P* = 0.012, OR = 4.649) were independently associated with therapy failure. Regular cleaning of the wound (*P* = 0.017, OR = 0.341) and sitz bath (*P* = 0.001, OR = 0.128) after I&D were independently associated with therapy success.

**Table 3:** Logistic regression analysis of risk factors for therapy failure after I&D for PAs.

	Univariate *P*	Multivariate^a^				
Factors		B	SE	Wald	*P*	OR
Sex	0.998					
History of PA	0.001	1.216	0.501	5.901	0.015	3.374
History of I&D	0.014	0.714	0.832	0.738	0.390	2.043
Sitz bath after I&D	0.016	−2.056	0.606	11.516	0.001	0.128
Regular cleaning of the wound after I&D	0.024	−1.075	0.450	5.700	0.017	0.341
Multilocal	0.003	1.537	0.610	6.338	0.012	4.649

I&D, incision and drainage alone; PA, perianal abscesses.

^a^ All significant factors in the univariate analysis (*P* < 0.05) were included in the multivariate analysis.

## 4 Discussion

Discussions in the medical fraternity continue to center around the management of PA in children. Both conservative treatment and surgical treatment have been reported with good outcomes (2, 4, 6, 8, 10, 12, 25). Surgery must be performed when an abscess is likely to spread or shows no sign of spontaneous perforation (4). Although there is another procedure that involves searching for the fistula and treating it at the same time (I&DF), which is also gaining in popularity, I&D remains the most common and parent-tolerated surgical treatment for PA in children in a real–world scenario. To the best of our knowledge, ours is the first study dedicated to identify risk factors for therapy failure after I&D for PAs in children. In this study, therapy failure occurred in 60 out of 172 (35%) treatments for PA by I&D. A history of PA and multilocal occurrence were the predictor factors for therapy failure before I&D. Regular cleaning of the wound and sitz bath after I&D were protective factors for therapy success. We hope that the results of our study are valuable to clinicians, as it can aid in making treatment plans, can be used to predict treatment efficacy, and can also be used to reasonably alleviate parental concerns.

The therapy failure rate of I&D ranged from 19.2% to 54% in the pediatric population described in other studies (10, 14, 15, 25, 27, 33). The therapy failure rate of I&D in our study is comparable to that in other studies (25, 33) but is higher than that of I&DF, for which the therapy failure rate has been reported to range from 0% to 12.1% (1, 3, 11, 33). Compared with I&DF, I&D remains more acceptable to both doctors and parents because of certain advantages. These include the avoidance of hospital admission and negating the risks of general anesthesia, thereby also reducing the anxiety of parents and the total outgoing financial cost (1, 4, 36). Most children with PA can be treated in outpatient clinics and do not require admission (13, 30). Although therapy failure after I&D exists, it can be managed easily by the parents of children without experiencing any physical or mental burden (30). For treating a patient with PA, it is not clinically appropriate for a surgeon to blindly recommend a certain type of surgery, whether it is I&D or I&DF. To choose an appropriate management strategy, it is important to identify the parameters that predict success or failure. Multimodal treatment may help further decrease the therapy failure rate (1).

In this study, we found that a history of PA increases the probability of therapy failure following I&D (*P* = 0.015, OR = 3.374. This is in agreement with the findings of Doerner et al. (33) and Boenicke et al. (37), who suggested that a history of PA was a predictor of surgical treatment failure, therapy failure caused by persisting infection (33). It was recommended that all patients who had recurrent episodes should be referred to specialists in colorectal surgery to reduce the risk of therapy failure. This recommendation was implemented by cautious probing when searching for a fistula, given the fact that a history of PA is a predictor of surgical treatment failure (33). In this study, we found that multilocal occurrence (*P* = 0.012, OR = 4.649) was independently associated with therapy failure, which was in agreement with the findings of Chang et al. (13) and Doerner et al. (33). This may be explained by the relative severity of precipitating factors, such as diarrhea, which would not have been alleviated or eliminated in time.

Adequate drainage and prevention of premature healing of the skin over a PA is a crucial goal of postoperative treatments after I&D (11, 30, 38). The guidelines for adults state that inadequate drainage is a risk factor for recurrent PA (18). After I&D, we routinely instruct parents to clean the wound by making their children visit our outpatient clinic or using a cotton swab at home every day or every 3 days. A sitz bath is also strongly recommended three times a day, especially after every defecation. The above measures can help with adequate drainage and prevention of premature healing of the skin over the PA. Other pediatric studies have also recommended the sitz bath for the management of PA after I&D (4, 10, 11, 30). Watanabe et al. instructed parents to keep the incision open by stretching the skin during each diaper change (30). However, many parents find it difficult to perform regular cleaning of the wound effectively because of the inconvenience caused by repeated dressing changes and the time consumption of repeated medical visits. Because young children struggle to cooperate with their parents during a sitz bath, parents often use a wet compress of KangFuXin instead. KangFuXin (*Periplaneta americana* extract) is a liquid preparation (39) that helps wound healing after I&D. In the multivariate analysis, we found that regular cleaning of the wound (*P* = 0.017, OR = 0.341) and sitz bath (*P* = 0.001, OR = 0.128) after I&D were independently associated with therapy success, which was in agreement with the data of Onaca et al. (40). In light of this, proper care measures after I&D should be emphasized to parents (11). Regular cleaning of the wound can prevent a premature healing of the skin and promote adequate drainage. A sitz bath can keep the wound clean, reduce wound bacteria, and also reduce inflammatory response caused by thermotherapy and pharmacotherapy.

The locations of abscesses were laterally distributed (70%) in our study, which was in agreement with other pediatric studies (3, 9, 12, 19, 23, 30, 36, 41). Chang et al. (13) also found that the location of an abscess was not a statistically significant factor for therapy efficacy, but Afsarlar et al. (29) found that the first and third quadrants of PA had the highest percentages of FIA development, significantly higher than that of the second and fourth quadrants. In our study, all treatment failures occurred in male patients, which was in agreement with other studies in the pediatric population (12, 14, 26). Some have regarded this phenomenon as presumptive evidence that PA in males occurs in relation to the perianal glands, with intestinal organisms being responsible for infection, and that in females, it is likely to be a superficial and localized infection (26, 30). Nix et al. found that fistula formation occurred only in those with intestinal bacterial infection (27).

Juth Karlsson et al. found that younger patients showed a higher recurrence rate compared with older patients after surgical treatment of PA (12). But in our study, age at I&D was not a significant factor for therapy efficacy, regardless of whether it was considered as a continuous variable or dichotomized into 1 or 2 years old. Younger age was also not associated with recurrent disease in other studies in the pediatric population (6, 13, 29). The effect of antibiotics on the therapy efficiency of I&D is controversial. Some researchers support the hypothesis that the use of antibiotics reduces treatment failure (17, 29), but this remains a topic of debate (12, 14, 33). We found that the use of antibiotics was not a significant factor for therapy efficacy. In our center, we did not use antibiotics routinely after I&D, unless patients presented with severe inflammation or systemic manifestation such as fever and irritability.

Different from adults (18), the indication and ideal timing for I&D of PAs in children remains unknown and controversial (37). Some recommend that PAs in children should be incised as soon as possible (30), while others recommend that I&D should be reserved only for patients with failed conservative management, a large abscess, or systemic signs of infection (3, 4, 12, 17, 34). Yet, abscesses have been removed or perforated spontaneously after conservative treatment in many patients without any systemic signs of infection (2, 4).

Our study has several strengths. First, it was the first study dedicated to identifying risk factors for therapy failure after I&D for PAs in children. Second, our sample size (172 treatments) was very large, which led to improved analysis and reduced bias to a certain extent, thereby increasing reliability and accuracy. Finally, we reviewed all outpatient children with PA treated by different doctors, which depicts real-life situations more accurately. The risk factors identified in our study for therapy failure after I&D are expected to be clinically useful.

Some limitations in our study need to be recognized. First, it is a single-center retrospective study, potentially limiting the generalizability of our results. Second, the factor of heterogeneity in daily clinical and surgical practice cannot be eliminated, because the treatment strategy mostly depends on the personal experiences of doctors, as well as the predilection of parents rather than established guidelines. A large number of surgeons may favor I&D over physicians and dermatologists (1). Perhaps parental anxiety and pressure motivate surgeons to perform I&D (22). Finally, we cannot provide specific information on the duration of treatment and on the results of auxiliary tests such as blood routine, pus culture, and B-mode ultrasonography. This is attributed to parents being unable to recall accurate dates post-event and also to the fact that our center does not routinely perform these tests, which have been mentioned in some literature as useful for evaluating patients (9, 23, 37).

Nonetheless, our findings are clinically useful for identifying the type of patients who suffer from a greater risk of therapy failure after I&D. More studies are required to ascertain the reproducibility of our findings because of the limitations reported in previous studies. We advocate a prospective, randomized study to clarify the efficiency of different treatment methods in the pediatric population and both the necessity and the ideal timing for surgery of a PA.

## 5 Conclusions

A history of PA and multilocal occurrence were predictor factors for therapy failure before I&D. Regular cleaning of the wound and sitz bath after I&D were protective factors for therapy success. Therefore, regular cleaning of the wound and sitz bath after I&D should be emphasized in all children with PAs, especially in those with a history of PA and multilocal occurrence.

## Data Availability

The original contributions presented in the study are included in the article/Supplementary Material, and further inquiries can be directed to the corresponding author.
